# Efficacy and safety of stereotactic radiotherapy on elderly patients with stage I-II central non-small cell lung cancer

**DOI:** 10.3389/fonc.2024.1235630

**Published:** 2024-05-13

**Authors:** Xiaoqin Ji, Bin Zhou, Hua Huang, Yong Wang, Wanrong Jiang, Jiasheng Wang, Wei Ding, Zhen Wang, Guanha Chen, Xiangdong Sun

**Affiliations:** ^1^ Department of Radiation Oncology, The First Affiliated Hospital of Nanjing Medical University, Nanjing, China; ^2^ Department of Radiation Oncology, Jinling Hospital, Affiliated Hospital of Medical School, Nanjing University, Nanjing, China; ^3^ Department of Outpatient clinic, Jinling Hospital, Affiliated Hospital of Medical School, Nanjing University, Nanjing, China

**Keywords:** NSCLC, stereotactic body radiation therapy, BMI, C-reactive protein, albumin

## Abstract

**Background:**

Many studies demonstrated the safety and efficacy of SBRT in the treatment of elderly patients with early-stage non-small cell lung cancer (NSCLC). However, those studies focused on patients with peripheral lung cancer. This study aimed to evaluate the clinical efficacy and toxicity of SBRT in elderly patients with stage I-II central NSCLC in single institution.

**Methods:**

From April 2009 to January 2020, a retrospective study was conducted on patients ≥ 65 years old with stage I-II NSCLC that was centrally localized and treated with SBRT at a single institution. Absolute C-reactive protein (CRP)/albumin ratio (CAR) and body mass index (BMI) recorded at pretreatment were analyzed. Endpoints included overall survival (OS), progression-free survival (PFS), cancer-specific death, noncancer-specific death, local progression (LP) and distant progression (DP).

**Results:**

Stereotactic body radiation treatment (SBRT) was administered to a total of 44 patients. The most common dose fractionation schedule was 60 Gy given in 5 fractions. The median PFS of the cohort was 31 months (95% CI, 19.47–42.53 months). The median OS of all patients was 69 months (95% CI, 33.8–104.2 months). The median time to noncancer-specific death was 54.5 months. The median time to cancer-specific death was 36 months. The cumulative incidences of cancer-specific death at 1 year, 5 years, and 10 years were 11.63% (95%CI, 4.2–23.23%), 42.99% (95%CI, 27.56–57.53%), and 65.94% (95%CI, 45.76–80.1%), respectively. pre-SBRT BMI of ≤ 22.77 (HR 4.60, 95% CI 1.84–11.51, P=0.001) and pre-SBRT CAR of ≤0.91 (HR 5.19, 95% CI 2.15–12.52, P<0.000) were significant predictors of higher OS on multivariable analysis. The median times to LP and DP were 10 months and 11 months, respectively. In terms of acute toxicity, grade 1 including cough (38.64%), radiation pneumonitis (29.55%), anemia (25%), and fatigue (20.45%) was often observed. There was no evidence of grade 4 or 5 acute toxicity. In terms of late toxicity, 2 patients developed grade 1 pulmonary fibrosis during follow-up.

**Conclusion:**

This study showed that SBRT can effectively control local tumor progression, and have acceptable toxicity for elderly patients with centrally located stage I-II NSCLC. Lower pre-SBRT BMI and lower pre-SBRT CAR were associated with a decreased risk of cancer-specific death.

## Introduction

Aging poses a significant social challenge for developed countries. In recent years, the aging population and improved screening methods have led to an increased detection rate of early lung cancer. More than two-thirds of lung cancer patients are aged 65 or older, and many have competing comorbidities. These patients have a low surgical tolerance rate due to organ degeneration. Approximately 20% of patients with stage I NSCLC is unable to undergo surgery because of poor general condition or comorbidities, such as chronic obstructive pulmonary disease and heart disease (1). For high-dose irradiation, SBRT focuses high-energy radiation beams on a certain limited lesion target area. This can cause irreversible biological damage, while normal tissues are spared or less irradiated. SBRT plays an important role in the treatment of early-stage NSCLC.

Existing studies have shown that the 2-year local control rates of SBRT for early inoperable NSCLC can reach 80%-97% (2, 3), and the overall survival rate and tumor-specific survival rate are comparable to those of surgery (4). Compared SBRT with lobectomy in 58 patients with operable stage I NSCLC, Chang et al. found that 3-year overall survival (OS) was 95% in the SBRT group and 79% in the surgery group (P=0.037) (5). One potential reason for the poor surgical survival may be non-cancer-related surgical deaths. This may be most relevant in older populations. Eguchi et al. conducted a retrospective analysis of 2,186 patients with pathologic stage I NSCLC who underwent curative-intent resection. They found that noncancer-specific mortality is a significant cause of death in patients over 75 years of age (6).

The use of SBRT as a treatment modality in early-stage NSCLC becomes more attractive for an aging population with increasing age-related comorbidities. This is due to similar rates of local tumor control across surgery and SBRT. However, the rate of morbidity and mortality of SBRT were lower than that of surgery. Trials have demonstrated the efficacy and safety of SBRT in treating elderly NSCLC patients (7–9).

Numerous studies have shown that SBRT can effectively reduce tumors in patients with early-stage NSCLC. However, the majority of the patients in those studies had peripheral lung cancer. The adverse reaction of radiation becomes the key limiting factor in central lung cancer, because there are many important tissues and organs in this area, such as trachea, bronchi, great vessels, esophagus, etc. It is necessary to balance tumor control and adverse reactions. Studies have shown that the closer the tumor to the bronchial tree, the higher the risk of SBRT-induced adverse reactions and non-tumor-related death (10). Regarding the application of SBRT in central lung cancer, important prospective studies mainly include RTOG 0813 and Lungtech studies. The RTOG 0813 study provided robust data on the safety and efficacy of a five-fraction SBRT regimen that was well tolerated and associated with a relatively low incidence of serious treatment-related toxicities (11).

Systemic inflammation and nutritional status, as measured by the systemic inflammatory response index (SIRI), prognostic nutrition index (PNI), neutrophil to lymphocyte ratio (NLR), platelet to lymphocyte ratio (PLR), and C-reactive protein (CRP)/albumin ratio (CAR), are associated with survival of many malignancies (12–16), including lung cancer (17–20). These markers are promising predictors of cancer prognosis due to their cost-effectiveness and ease of detection. For example, elevated baseline lactate dehydrogenase (LDH) levels and derived NLR were associated with poorer survival in patients with metastatic NSCLC regardless of treatment (21).

However, there are few studies on the serum inflammatory markers and/or nutritional markers in predicting prognosis after SBRT of elderly stage I-II central NSCLC. Therefore, this study evaluated the efficacy and safety of SBRT in elderly patients with stage I-II central NSCLC.

## Methods

### Patients

A retrospective analysis was performed on 44 patients aged ≥65 years with centrally located stage I-II NSCLC. Most of these patients were medically inoperable. They underwent SBRT at Jinling Hospital from April 2009 to January 2020. Patient inclusion criteria were as follows (1): Diagnosis is confirmed by histology or typical clinical presentation and positron emission tomography/computed tomography (PET/CT) (2); Clinical stage I-II was assessed by whole-body imaging examination based on the eighth edition of the TNM staging system of the American Joint Committee on Cancer (3); Imaging showed a central lung cancer, which is defined as the tumor invasion within 2 cm in all directions of the proximal bronchial tree or PTV adjacent to the mediastinal/pericardial pleura (**Supplementary Figure 1**). Patient exclusion criteria were as follows: (1) Patients with previous history of malignancy; (2) Clinical stage III-IV; (3) Small cell lung cancer patients. Blood samples were routinely collected within 1 week before SBRT. The calculation formulas of NLR, PLR, PNI and SIRI were described in our previous study (22). This study was performed in accordance with the Declaration of Helsinki (as revised in 2013). This study was approved by the Institutional Review Board of Jinling hospital (No. 2023NZKY-034–02).

### Stereotactic body radiotherapy

In this study, CyberKnife (Accuray Incorporated, Sunnyvale, CA, USA) was used for SBRT. 1–3 gold fiducials were implanted into the lesion under CT guidance. The CT positioning scan was performed 7 days later. The movement of fiducials was monitored via respiration synchronous tracking (Synchrony). 10 cases of lesions close to the thoracic spine were treated using the XSight spine tracking method. The patient was in a supine position and fixed with a vacuum pad during chest CT simulation positioning. The CT scans encompassed the entire circumference of the body contour with coverage from 15 cm above the lesion to 15 cm below the lesion. CT slice was 1 mm thick. Based on the tumor volume, the gross tumor volume (GTV) was calculated. Clinical tumor volume (CTV) was equal to that of GTV. GTV was expanded 0–8 mm to form the planning target volume (PTV). The central lung cancer SBRT limits for normal tissues and organs are based on the RTOG 0813 study, the MD Anderson Center experience, and the Radiation Therapy Oncology Group (RTOG)/NRG SBRT protocol (4, 23, 24) (**Supplementary Table 1**). The CyberKnife treatment planning, once confirmed and signed by both the physician and physicist, was transmitted to the CyberKnife control platform for execution. The technician thoroughly examined various parameters to ensure their accuracy before initiating the treatment process. SBRT was performed once a day, five days per week.

### Systemic therapy

Patients with stage I NSCLC did not receive systemic treatment. The chemotherapy regimen for stage II NSCLC was based on platinum-based doublet chemotherapy. The median number of chemotherapy cycles was 4, with a range of 2 to 10.

### Follow-up and statistics

Follow-up assessments were conducted 1 month after SBRT, and every 3 months for the first three years. From the fourth to fifth year, assessments were performed every 6 months. Then, there was an annual follow-up after five years. The National Cancer Institute’s Common Terminology Criteria for Adverse Events, version 5.0 (CTCAE), was used to assess toxicity. Acute toxicity was defined as an event within 90 days of radiation therapy initiation. Events occurring more than 90 days after the start of SBRT were considered as the late toxicity.

The endpoints of this study were progression-free survival (PFS), overall survival (OS), cancer–specific death, noncancer-specific death, local progression, and distant progression. The causes of death were divided into cancer specific and noncancer specific. PFS was defined as the time from initiation of SBRT to progression at any site. OS was defined as the time from the start of SBRT to the last follow-up or death. Cancer–specific death was defined as death from progressive disease associated with lung cancer. Noncancer-specific death was defined as death from specific causes other than the malignant disease. Local progression refers to tumor recurrence within the irradiated volume, defined as the time from initiation of SBRT to local progression. Distant progression was defined as the time from initiation of SBRT to the appearance of new lesions outside the target volume.

The associations between factors and the risk of each cause of death, local progression and distant progression were assessed by competing risks analysis. A death without interest event is a competent event. Cumulative incidence functions were estimated using competing hazards analysis (Gray’s test). X-tile soft was used to determine the optimal cutoff for continuous variables. Univariate analysis of PFS and OS was performed using the Kaplan-Meier method. Log-rank was used as a statistical test method for Kaplan-Meier survival analysis. Only variables with P less than 0.05 in the univariate analysis were studied in multivariate analysis. The cox proportional hazards model was utilized for multivariate analysis. All statistical analyzes were performed using SPSS 24.0 statistical software and R 4.3.0, and a P value less than 0.05 was statistically significant.

## Results

### Patient characteristics

A total of 44 central stage I-II NSCLC patients aged ≥65 years, who underwent SBRT were included in this study (**Table 1**). The patients’ ages ranged from 65 to 90 years (median age was 76 years). Of these patients, 40 (90.91%) were men, and 4 (9.09%) were women. The Eastern Cooperative Oncology Group performance status (ECOG) scores of patients were between 0 and 2. The primary symptoms were cough (n=26, 59.1%), hemoptysis (n=14, 31.8%), chest tightness (n=8, 18.2%), and chest pain (n=5, 11.4%). 7 patients (15.91%) refused the biopsy due to concerns about toxicity. A total of 41 patients (93.2%) were medically inoperable. 3 patients (6.8%) refused primary surgery.

**Table 1:** Patients’ characteristics.

Characteristics	No. of patients (% of total)
Patients	44 (100)
SBRT indication	
Refused surgery	3 (6.8)
Medically inoperable	41 (93.2)
Age (years), median (range)	76 (65-90)
Gender	
Female	4 (90.91)
Male	40 (9.09)
BMI (kg/m^2^), median (range)	22.53 (15.57-27.82)
Smoking history	
No	18 (40.91)
Yes	26 (59.09)
Years of smoking	25 (0-60)
Number of cigarettes smoked per day	15 (0-60)
Smoking index	450 (0-2400)
Performance status	
0	2 (4.54)
1	32 (72.73)
2	10 (22.73)
aCCI	
≤5	19 (43.18)
>5	25 (56.82)
Hypertension	
No	24 (54.55)
Yes	20 (45.45)
Diabetes	
No	41 (93.18)
Yes	3 (6.82)
Coronary heart disease	
No	41 (93.18)
Yes	3 (6.82)
Chronic obstructive pulmonary disease	
No	33 (75)
Yes	11 (25)
Symptom	
Coughing	26 (59.09)
Hemoptysis	14 (31.82)
Chest tightness	8 (18.18)
Chest pain	5 (11.36)
No	10 (22.73)
Tumor near the central structures	
Aorta	14 (31.82)
Heart	7 (15.91)
Esophagus	4 (9.09)
Trachea/central bronchial airways	19 (43.18)
T-stage	
T1	8 (18.18)
T2	24 (54.55)
T3	12 (27.27)
N-stage	
N0	41 (93.18)
N1	3 (6.82)
TNM-stage	
I	19 (43.18)
IIA	10 (22.73)
IIB	15 (34.09)
Histologic type	
Squamous cell carcinoma	33 (75)
Adenocarcinoma	4 (9.09)
Unknown	7 (15.91)
Treatment before SBRT	
Chemotherapy	10 (22.73)
Erlotinib	1 (2.27)
None	33 (75)
Treatment after SBRT	
Chemotherapy	1 (2.27)
Traditional Chinese medicine	2 (4.55)
None	41 (93.18)
Chemotherapy cycles, median (range)	4 (2-10)
Time from diagnosis to SBRT (days), median (range)	34.5 (6-754)
Blood samples before SBRT	
SIRI, median (range)	1.415 (0.39-17.54)
NLR, median (range)	3.005 (1.26-18.46)
PLR, median (range)	148.25 (50.84-513.05)
PNI, median (range)	47.55 (32.15-59.25)
CAR, median (range)	0.13 (0.01-3.41)
LCR, median (range)	0.235 (0.01-4.18)
RBC (10^12/L), median (range)	4.14 (2.24-5.08)
Hb (g/L), median (range)	122.5 (69-155)

SBRT, stereotactic body radiotherapy; BMI, body mass index; aCCI, Age-adjusted Charlson Comorbidity Index; SIRI, systemic inflammation response index; NLR, neutrophil to lymphocyte ratio; PLR, platelet to lymphocyte ratio; PNI, prognostic nutritional index; CAR, C-reactive protein to albumin ratio; LCR, lymphocyte to C-reactive protein ratio; RBC, red blood cell count; Hb, hemoglobin.

### Treatment characteristics

A total of 44 patients with 47 tumor lesions were treated by SBRT. Among them, 3 patients received SBRT to 3 ipsilateral hilar regional lymph nodes. The median time from the diagnosis of NSCLC to SBRT was 34.5 days (range of 6–754 days). Of these patients, 10 patients underwent chemotherapy and 1 patient underwent Erlotinib before SBRT. After SBRT, two patients received traditional Chinese medicine, while one patient underwent systemic chemotherapy. The most common chemotherapy regimen was platinum-based chemotherapy (**Table 1**).

The GTV volume ranged from 9.2 to 122.4cc, with a median of 24.2cc. The PTV volume ranged from 7.8 to 282.4cc, with a median of 66.8cc. The range of PTV coverage was 62.04% to 98.92%, with a median of 92.0%. The duration of treatment was 3–8 days. The median prescribed dose was 52.5 gray (Gy) (range of 42–60 Gy), given in 3 to 6 fractions. α/β was assumed to be 10, and median BED_10_ was 122.4 Gy (range of 71.4–180 Gy). The median isodose of prescriptions was 76.0%. SBRT planning and delivery variables were summarized in **Table 2**.

**Table 2:** SBRT planning and delivery variables (N=44 patients).

Variables	Number
Prescription dose, most common, Gy	60
Fraction dose, most common, Gy	12
BED_10_, most common, Gy	132
10Gy x 6, no. of patients	1
9Gy x 6, no. of patients	1
8Gy x 6, no. of patients	2
7Gy x 6, no. of patients	1
12Gy x 5, no. of patients	14
10Gy x 5, no. of patients	13
9Gy x 5, no. of patients	3
15Gy x 4, no. of patients	1
12Gy x 4, no. of patients	1
20Gy x 3, no. of patients	4
18Gy x 3, no. of patients	1
17Gy x 3, no. of patients	1
16Gy x 3, no. of patients	1
Min dose to PTV, median (range), Gy	38 (20.9-55.6)
Max dose to PTV, median (range), Gy	69.3 (52.5-98.6)
Min dose to GTV, median (range), Gy	42.4 (21.4-62.9)
Max dose to GTV, median (range), Gy	69.3 (40.72-98.4)
Target size, median (range), cm	5.15 (2.1-9.2)
PTV volume, median (range), cc	66.8 (7.8-282.4)
GTV volume, median (range), cc	45.8 (3.0-219.8)
PTV coverage (%), median (range)	91.995 (62.04-98.92)
GTV coverage (%), median (range)	98.30 (72.47-100)
Prescription isodose line (%), median (range)	76 (61-86)
HI, median (range)	1.3 (1.2-1.6)
CI, median (range)	1.13 (1.01-1.36)
nCI, median (range)	1.265 (1.14-1.71)

PTV, planning tumor volume; GTV, gross tumor volume; BED, biological effective dose; HI, homogeneity index; CI, conformity index; nCI, new conformity index.

### Survival analysis

#### Progression free survival and overall survival

**Figure 1:**
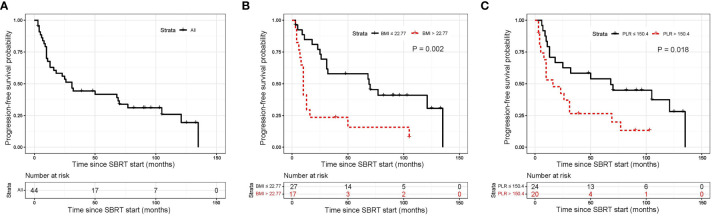
Kaplan-Meier curves for progression free survival. **(A)** shows of all patients; **(B)** shows BMI before SBRT; **(C)** shows PLR before SBRT. SBRT, stereotactic body radiotherapy.

The median follow-up period for all 44 patients was 110 months (95% CI, 88.97–131.03 months). The median PFS was 31 months (95% CI, 19.47–42.53 months; **Figure 1A**). The 1-year, 5-year, and 10-year PFS rates were 67.5% (95%CI, 51.39–79.31%), 41.6% (95%CI, 26.83–55.78%) and 25.8% (95%CI, 12.48–41.48%), respectively (**Table 3**). BMI and PLR were significantly associated with PFS in univariate analysis (**Supplementary Table 2**). In multivariable analysis, BMI ≤ 22.77 (HR 3.14, 95% CI 1.48–6.67, P=0.003; **Figure 1B**), and PLR ≤ 150.36 (HR 2.44, 95% CI 1.15–5.16, P=0.020; **Figure 1C**) were significantly associated with longer PFS (**Table 4**).

**Table 3:** Survival rate and cause of mortality.

	1-year rate (%) (95% CI)	2-year rate (%) (95% CI)	3-year rate (%) (95% CI)	5-year rate (%) (95% CI)	10-year rate (%) (95% CI)
PFS	67.52 (51.39-79.31)	55.88 (39.91-69.16)	44.24 (29.20-58.25)	41.63 (26.83-55.78)	25.84 (12.48-41.48)
OS	86.09 (71.63-93.50)	81.44 (66.69-90.66)	62.66 (46.44-75.21)	50.08 (34.18-64.05)	24.34 (11.00-40.47)
Cancer-specific mortality	11.63 (4.20-23.23)	16.29 (7.07-28.85)	30.42 (17.33-44.56)	42.99 (27.56-57.53)	65.94 (45.76-80.10)
Noncancer-specific mortality	2.27 (0.17-10.49)	2.27 (0.17-10.49)	6.93 (1.76-17.15)	6.93 (1.76-17.15)	9.73 (2.99-21.30)
Local progression	11.59 (4.18-23.15)	16.24 (7.05-28.77)	20.90 (10.22-34.15)	–	–
Distant progression	11.63 (4.20-23.24)	16.29 (7.07-28.86)	18.61 (8.62-31.57)	–	–

PFS, progression-free survival; OS, overall survival.

**Table 4:** Multivariable analysis of PFS, OS and cancer-specific death after SBRT.

Variables	PFS			OS			Cancer-specific death		
	HR	95%CI	P value	HR	95%CI	P value	SHR	95%CI	P value
BMI (kg/m^2^)			0.003			0.001			0.001
≤22.77	Ref			Ref			Ref		
>22.77	3.14	1.48-6.67		4.75	1.92-11.72		4.60	1.84-11.51	
Smoking Index			–			0.004			–
≤1100	–	–		Ref			–	–	
>1100	–	–		3.71	1.52-9.04		–	–	
PLR			0.020			–			–
≤150.36	Ref			–	–		–	–	
>150.36	2.44	1.15-5.16		–	–		–	–	
CAR			–			–			0.000
≤0.91	–	–		–	–		Ref		
>0.91	–	–		–	–		5.19	2.15-12.52	

PFS, progression-free survival; OS, overall survival; BMI, body mass index; PLR, platelet to lymphocyte ratio; CAR, C-reactive protein to albumin ratio.

**Figure 2:**
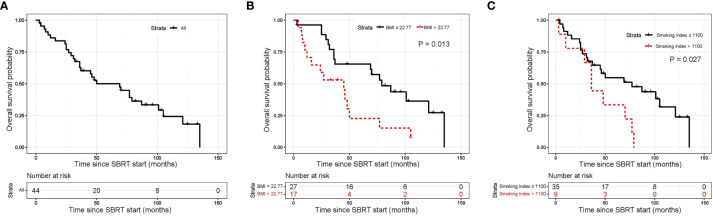
Kaplan-Meier curves for overall survival. **(A)** shows of all patients; **(B)** shows BMI before SBRT; **(C)** shows pre-SBRT smoking index. SBRT, stereotactic body radiotherapy.

The median OS was 69 months (95% CI, 33.8–104.2 months; **Figure 2A**). The 1-year, 5-year, and 10-year OS rates of all patients were 86.1% (95%CI, 71.63–93.50%), 50.1% (95%CI, 34.18–64.05%) and 24.3% (95%CI, 11.00–40.47%), respectively (**Table 3**). In univariate analysis, BMI, smoking Index, performance status, PLR and CRP/Alb were significantly associated with OS (**Supplementary Table 2**). In multivariable analysis, BMI ≤ 22.77 (HR 4.75, 95% CI 1.92–11.72, P=0.001; **Figure 2B**), and smoking index ≤ 1100 (HR 3.71, 95% CI 1.52–9.04, P=0.004; **Figure 2C**) were significantly associated with longer OS (**Table 4**).

#### Cause of death

**Figure 3:**
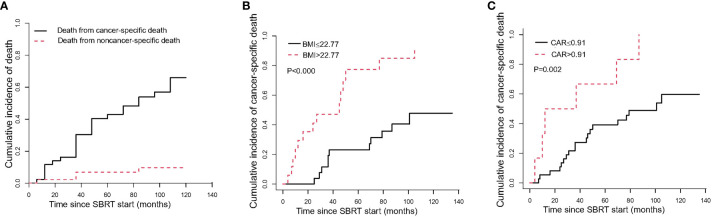
The cumulative incidence curve of death after SBRT. **(A)** shows the probability of each competing event in the entire cohort; **(B)** shows the cumulative incidence curve of cancer-specific death by pre-SBRT BMI level; **(C)** shows the cumulative incidence curve of cancer-specific death by pre-SBRT CAR level. CAR, C-reactive protein to albumin ratio.

The median time to cancer-specific death was 36 months. The cumulative incidence of cancer-specific death at 1 year, 5 years, and 10 years were 11.63% (95%CI, 4.2–23.23%), 42.99% (95%CI, 27.56–57.53%), and 65.94% (95%CI, 45.76–80.1%), respectively (**Table 3**, **Figure 3A**). In univariable analyses, predictors for cancer specific death were comorbidity, BMI, PNI and CAR (**Supplementary Table 3**). In multivariable analysis, BMI ≤ 22.77 (HR 4.60, 95% CI 1.84–11.51, P=0.001; **Figure 3B**) and ≤0.91 CAR (HR 5.19, 95% CI 2.15–12.52, P<0.000; **Figure 3C**) were independent predictors of cancer specific death (**Table 4**).

There were 6 non-cancer specific deaths due to exacerbations of chronic obstructive pulmonary disease (3 patients), cardiac insufficiency (2 patients), and intestinal obstruction (1 patient). The median time to noncancer-specific death was 54.5 months. The cumulative incidences of noncancer-specific death at 1 year, 5 years, and 10 years were 2.27% (95%CI, 0.17–10.49%), 6.93% (95%CI, 1.76–17.15%), and 9.73% (95%CI, 2.99–21.30%), respectively (**Table 3**, **Figure 3A**).

#### Local progression and distant progression

**Figure 4:**
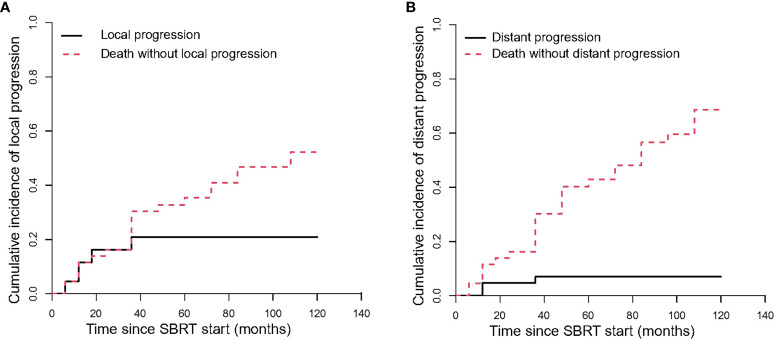
The Cumulative incidence curve of progression after SBRT. **(A)** shows the local progression; **(B)** shows the distant progression.

The median time to local progression (LP) was 10 months. The 1-year, 2-year and 3-year cumulative incidences of LP were 11.59% (95%CI, 4.18–23.15%), 16.24% (95%CI, 7.05–28.77%), and 20.9% (95%CI, 10.22–34.15%), respectively (**Table 3**, **Figure 4A**). We cannot perform univariate analysis because of the small number of events.

The median time to distant progression (DP) for all patients was 11 months. The cumulative incidences of DP were 4.65% (95%CI, 0.83–14.06%) and 6.98% (95%CI, 1.77–17.26%) at 1 year and 3 years, respectively (**Table 3**, **Figure 4B**).

### Toxicity

Overall, the treatment was well tolerated. All patients received complete SBRT. There were no therapy-related deaths. In terms of acute toxicity, grade 1 including coughing (38.64%), radiation pneumonitis (29.55%), anemia (25%), and fatigue (20.45%) were often observed. The incidence of grade 3 non-hematological toxicity was 9.1%. For overall hematological toxicity, cases of grades 2 and 3 accounted for 20.4% and 6.8% of the total cases, respectively. No acute toxicities of grade 4 or 5 were observed. In terms of late toxicity, 2 patients developed grade 1 pulmonary fibrosis. The observed SBRT-related toxicity events were summarized in **Table 5**.

**Table 5:** Acute and late toxicities (N = 44 patients).

Toxicity	Grade 1 (%)	Grade 2 (%)	Grade 3 (%)	Grade 4 (%)	Grade 5 (%)
Acute					
Hematological					
Anemia	11 (25)	5 (11.36)	3 (6.82)	0 (0)	0 (0)
Leukopenia	3 (6.82)	3 (6.82)	0 (0)	0 (0)	0 (0)
Neutropenia	2 (4.55)	1 (2.27)	0 (0)	0 (0)	0 (0)
Thrombocytopenia	2 (4.55)	0 (0)	0 (0)	0 (0)	0 (0)
Non-hematological					
Cough	17 (38.64)	5 (11.36)	0 (0)	0 (0)	0 (0)
Hemoptysis	3 (6.82)	0 (0)	0 (0)	0 (0)	0 (0)
Hoarseness	4 (9.09)	0 (0)	0 (0)	0 (0)	0 (0)
Dyspnea	2 (4.55)	1 (2.27)	0 (0)	0 (0)	0 (0)
Chest pain	2 (4.55)	0 (0)	0 (0)	0 (0)	0 (0)
Dysphagia	2 (4.55)	1 (2.27)	0 (0)	0 (0)	0 (0)
Fatigue	9 (20.45)	4 (9.09)	0 (0)	0 (0)	0 (0)
Anorexia	6 (13.64)	3 (6.82)	1 (2.27)	0 (0)	0 (0)
Radiation pneumonitis	13 (29.55)	7 (15.91)	3 (6.82)	0 (0)	0 (0)
Esophagotracheal fistula	0 (0)	0 (0)	0 (0)	0 (0)	0 (0)
Late					
Radiation fibrosis	2 (4.55)	0 (0)	0 (0)	0 (0)	0 (0)
Stenosis	0 (0)	0 (0)	0 (0)	0 (0)	0 (0)
Cardiac	0 (0)	0 (0)	0 (0)	0 (0)	0 (0)

## Discussion

SBRT has been widely utilized in the treatment of inoperable early-stage peripheral lung cancer and has demonstrated promising outcomes. The local control rate exceeds 90%, the 3-year overall survival rate is over 70%, and there are no adverse reactions greater than grade 3 (25, 26). However, the application of SBRT in central lung cancer has been considered a no-fly zone due to its inherent challenges and complexities. With the advancement of radiotherapy technology and the availability of high-quality imaging, SBRT has been increasingly utilized in the treatment of central lung cancer, yielding favorable outcomes. This retrospective study demonstrated that SBRT provides favorable survival, tumor control and safety for central early-stage elderly NSCLC patients. The efficacy of SBRT for centrally situated lung tumors has been demonstrated in several trials.

Most studies shown that SBRT achieved excellent local control. A four-year retrospective study reviewed 31 consecutive central early-stage NSCLC patients who were treated with SBRT (BED 100–119 Gy in 4-10 fractions) (27). The incidence of local disease recurrence at 3 years and 5 years were 11.7% and 21.5%, respectively. Zhao et al. (28) performed a retrospective analysis of 98 patients who underwent SBRT at 60 Gy in 8 fractions to central and ultra-central lung cancers. They found that local control rates were 97.8%, 93.7% and 84.5% at 1-, 2- and 3-year, respectively. A phase I/II study of NRG Oncology/RTOG 0813 summarized 120 patients with staged T1 to 2 (≤ 5 cm) N0M0 centrally located NSCLC who were treated by SBRT (10–12 Gy/fx*5 fx). The 2-year local control rates in the 11.5 Gy/fx and 12.0 Gy/fx cohorts were 89.4% and 87.9%, respectively (11). Another systematic review enrolled 315 early-stage NSCLC patients with 563 central lung lesions for SABR (stereotactic ablative radiotherapy) (29). When the prescribed bioequivalent dose was higher than 100 Gy, the local control rate was higher than 85%. In addition, Roach et al. (30) showed that 2-year local control rate was 85% (55Gy/5fx). 1-, 2-, and 3-year cumulative incidences of LF in this study were 11.59%, 16.24%, and 20.9%, respectively. They were slightly lower. Our modest rates of local control are probably a result of the larger tumors and elderly patients. Modh et al. (31) found that gross tumor volume was significantly associated with LF.

In this study, we analyzed cancer-specific and non-cancer-specific deaths. Noncancer-specific death was higher because older cohorts had more comorbidities. This underscores the clinical significance of assessing non-cancer-specific death as a competing event in elderly patients. In this study, most non-cancer-specific deaths were attributable to cardiorespiratory diseases.

There are several studies on central lung cancer showed that SBRT achieved good OS rates. Sun et al. (27) showed that the 3- and 5-year OS rates were 85.3% and 68.4%, respectively. NRG Oncology/RTOG 0813 trial showed that 2-year OS rates in the 11.5 and 12.0 Gy/fx cohorts were 67.9% and 72.7%, respectively (11). Roach et al. (30) found that the 2-year overall survival for patients with centrally located was 43% in a prospective phase I/II trial, early-stage NSCLC receiving SBRT. In this study, 2-year, 3-year, and 5-year OS rates were 81.44%, 62.66% and 50.08%, respectively. Cumulative incidences of cancer-specific death at 2-year, 3-year, and 5-year were 16.29%, 30.42%, and 42.99%, respectively. Although our study exclusively enrolled the elderly patient population, OS was unexpectedly longer than those reported in studies that also included younger patients. For elderly patients with comorbid conditions such as severe heart disease, hypertension, diabetes, and other comorbidities, SBRT emerges as a superior treatment option. This finding holds significant implications for the field and supports the utilization of SBRT in the treatment of elderly patients with early stage central NSCLC.

More and more evidence showed that systemic inflammation plays a role in tumor progression and survival of cancer patients (32). C-reactive protein (CRP), a routinely measured marker of inflammation, is increasingly expressed in tumors. Furthermore, CRP is an important prognostic indicator in a number of malignancies, including pancreatic cancer (33), urological cancer (34), hepatocellular carcinoma (35), and NSCLC (36–38). In addition, malnutrition is common in cancer patients due to a marked increase in energy expenditure resulted from increased tumor metabolism (39). Albumin reflects nutritional status and response to inflammation and is associated with treatment outcomes in cancer patients. In NSCLC patients, serum albumin is an important prognostic factor for survival (40, 41). Therefore, some studies used the combination of CRP and albumin to predict the prognosis of cancer patients. In a study of 104 patients with cT4b esophageal squamous cell carcinoma, Sohda et al. found that the high C-reactive protein/albumin group had a significantly lower prognosis than the low C-reactive protein/albumin group in terms of disease-specific survival and overall survival group (42). Patil et al. found that CRP/Albumin ratio was a useful predictor of overall survival and recurrence in patients with clear cell renal cell carcinoma (16). The CRP/Alb ratio can predict the prognosis of NSCLC patients (43–45). Yang et al. found that elevated CRP/Alb ratio decreased survival of these patients in the study of 387 patients with primary NSCLC (46). Similarly, our study showed that higher CRP/Albumin ratio was independently associated with higher cancer related mortality.

More and more data showed that a link between greater body mass index (BMI) and better outcomes for patients with advanced malignancies and other acute or chronic disorders (47–49).. Both muscle and fat reserves are important in advanced cancer patients, who are often treated relatively aggressively. However, the mechanism of protective is unclear. In this study, patients were in early-stage NSCLC. Due to their older age and comorbidities, the relatively weak treatment intensity, patients did not show the survival advantage of higher BMI. In this study, a higher BMI was associated with a lower cancer-specific mortality in elderly patients with NSCLC. This difference can be explained by the association of high BMI with greater cardiorespiratory load and chronic disease (type 2 diabetes or heart disease). The cancer-related mortality was high in patients with comorbidities in the univariate analysis.

For early stage NSCLC, SABR may be better tolerated than surgery. In the study of Chang et al. (5), they compared the efficacy of SBRT with lobectomy for operable stage I NSCLC. Preliminary results showed that SBRT has better efficacy and lower toxicities than surgery. However, the treatment of early central NSCLC by SBRT may cause severe radiation damages, because the target area is close to the important organs of the mediastinum (50, 51). Therefore, it is more prudent to carry out SBRT technology in early central NSCLC than in peripheral NSCLC. Early-stage lung cancer with a target margin close to the bronchi may cause fatal hemoptysis and severe dyspnea after SBRT. Li et al. (52) reported that 82 patients with clinically challenging early stage or isolated recurrent biopsy-confirmed NSCLC treated with SABR using 70 Gy in 10 fractions. The most common toxicities were radiation pneumonitis (14.6% grade 2, 2.4% grade 3) and chest wall pain (1.2% grade 3). However, one patient with bronchial tree tumor invasion died of hemoptysis. Mou et al. (53) found that 14.4% of 132 patients with central lung tumors treated with SBRT had grade 3 or higher pneumonia. In this study, 6.82% of patients had grade 3 radiation pneumonitis. Upon hospitalization, these patients exhibited significant improvement after receiving symptomatic treatments, including oxygen inhalation, dexamethasone, and cough medicines. For those elderly and long-term smoking patients, radiation pneumonitis above grade 2 may seriously affect the patient’s prognosis and quality of life. Therefore, although SBRT can be applied to early central NSCLC, it is necessary to protect the organs at risk and avoid the occurrence of severe treatment-related toxicity. Senthi et al. (29) found that treatment-related mortality was 2.7% overall, and grade 3 or 4 toxicities occurred in less than 9% of patients. In a study of RTOG 0813 (11), the maximum tolerated dose of 12.0 Gy/fx was associated with a dose-limiting toxicity of 7.2%. According to the study of Haasbeek et al. (54), Zhao et al. (28), no grade 4–5 toxicities occurred in patients with central lung cancer treated with SBRT to 60Gy in 8 fractions. They found that most patients experienced CTCAE grade 1–2 acute toxic events, and most symptoms were transient and resolved with conservative management. Late toxicities were reported in only 2 patients with grade 1 pulmonary fibrosis. This study showed that elderly patients with centrally located lung lesions were well tolerated by SBRT.

However, there are some limitations in this single-center retrospective study. The single-institution, retrospective nature may limit the generalizability of the findings. In addition, the number of patients was small, which may lead to statistical bias, selection bias and unmeasured confounders. Furthermore, some patients with NSCLC were clinically diagnosed. Therefore, the puncture biopsy should be carried out as far as possible before radiotherapy to clarify the pathology. This study included only patients who underwent SBRT, without valid comparators. Moreover, patients received a variety of SBRT dose regimens. In future study, it is imperative to conduct multicenter trials with larger patient cohorts, appropriate control groups, and consistent treatment regimens to elucidate the specific benefits of SBRT.

Our findings, which focus on the novel and significant impact of SBRT in the treatment of NSCLC among elderly patients with centrally located tumors, hold particular relevance in the broader context of lung cancer management. This patient subset often faces unique challenges due to their advanced age and tumor location, which can limit treatment options and impact prognosis. By demonstrating the efficacy and safety of SBRT in this population, our study offers a promising new approach that may improve outcomes and quality of life for these patients. In conclusion, SBRT is an alternative local treatment for elderly central early-stage NSCLC that can improve LC rates and survival, and is well tolerated without serious toxicities.

## Data availability statement

The raw data supporting the conclusions of this article will be made available by the authors, without undue reservation.

## Ethics statement

The studies involving humans were approved by the Institutional Review Board of Jinling hospital. The studies were conducted in accordance with the local legislation and institutional requirements. The ethics committee/institutional review board waived the requirement of written informed consent for participation from the participants or the participants’ legal guardians/next of kin because we reviewed the medical records.

## Author contributions

XJ: Conception and design, Manuscript writing, Final approval of manuscript. BZ: Data analysis and interpretation, Manuscript writing, Final approval of manuscript. HH: Collection and assembly of data, Data analysis and interpretation, Manuscript writing, Final approval of manuscript. YW: Conception and design, Manuscript writing, Final approval of manuscript. WJ: Conception and design, Manuscript writing, Final approval of manuscript. JW: Manuscript writing, Final approval of manuscript. WD: Data analysis and interpretation, Manuscript writing, Final approval of manuscript. ZW: Collection and assembly of data, Manuscript writing, Final approval of manuscript. XS: Conception and design, Manuscript writing, Final approval of manuscript. GC: Collection and assembly of data, Manuscript writing, Final approval of manuscript.

## Supplementary material

Supplementary Figure 1 CT images of several typical central lung cancer patients.
