# Supplementary material for: Efficacy and safety of stereotactic radiotherapy on elderly patients with stage I-II central non-small cell lung cancer
Source: Front Oncol. 2024 May 13;14:1235630. doi: 10.3389/fonc.2024.1235630 (PMC11128597; doi:10.3389/fonc.2024.1235630)
Supplement: Supplementary file 1 [file Table_1.docx]

| Supplementary table 1. OAR dose constraints applied for five fraction SBRT in this study. | | |
| --- | --- | --- |
| Normal tissue | Volume | Max dose |
| Total lung | Vtot - V_12.5_ >1500 cc* |  |
|  | Vtot - V_13.5_ >1000 cc |  |
|  | V5≤30% |  |
|  | V20≤12% |  |
| Trachea and large bronchus^※^ | V_16_ < 4 cc | D_max_≤38 Gy |
| Bronchus-smaller airways | V_21_ < 0.5 cc | D_max_≤33 Gy |
| Great vessel | V_47_<10 cc | D_max_≤53 Gy |
| Esophagus^※^ | V_19.5_ <5 cc | D_max_≤35 Gy |
| Heart | V_32_<15 cc | D_max_≤38Gy |
| Rib | V_35_ <1 cc | D_max_≤43Gy |
| Brachial plexus | V_30_<3 cc | D_max_≤30.5 Gy |
| Spinal cord | V_20_ < 0.35 cc | D_max_≤25 Gy |
| Chest wall | V_30_<30 cc | D_max_≤39.5 Gy |
| OAR: the organs at risk; V_x_: volume of tissue exposed to x Gy or more; D_max_: maximum dose; *The total volume of the lung minus the volume exposed to 12.5Gy was greater than 1500cc; ^※^Avoid circumferential irradiation. | | |
