# Supplementary material for: Efficacy and safety of stereotactic radiotherapy on elderly patients with stage I-II central non-small cell lung cancer
Source: Front Oncol. 2024 May 13;14:1235630. doi: 10.3389/fonc.2024.1235630 (PMC11128597; doi:10.3389/fonc.2024.1235630)
Supplement: Supplementary file 2 [file Table_2.docx]

| Supplementary table 2 Univariate analysis of factors affecting PFS and OS. | | | | | | | | |
| --- | --- | --- | --- | --- | --- | --- | --- | --- |
| Variables | PFS | | | |  | OS | | |
|  | Median PFS (95%CI) in months | | | P value |  | Median OS (95%CI) in months | | P value |
| Age (years)  ≤80  >80 |  | |  | 0.992 |  |  |  | 0.414 |
|  | 26 (0.00-82.34) | | |  |  | 70 (31.40-108.60) | |  |
|  | 31 (8.83-53.18) | | |  |  | 32 (8.44-55.56) | |  |
| Gender | | | | 0.801 |  |  |  | 0.793 |
| Female | 10 (-) | | |  |  | 12 (-) | |  |
| Male | 31 (20.03-41.97) | | |  |  | 69 (34.47-103.53) | |  |
| BMI (kg/m^2^) | |  |  | 0.002 |  |  |  | 0.013 |
| ≤22.77 | 69 (17.37-120.63) | | |  |  | 79 (54.44-103.56) | |  |
| >22.77 | 10 (8.01-11.99) | | |  |  | 45 (19.31-70.69) | |  |
| Smoking index |  | |  | 0.120 |  |  |  | 0.027 |
| ≤1100 | 32 (0.00-69.15) | | |  |  | 77 (27.43-126.57) | |  |
| >1100 | 25 (4.55-45.45) | | |  |  | 36 (25.77-46.23) | |  |
| Performance status |  | |  | 0.078 |  |  |  | 0.029 |
| 0/1 | 32 (0.00-78.66) | | |  |  | 77 (41.17-112.83) | |  |
| 2 | 18 (3.54-32.46) | | |  |  | 36 (15.75-56.25) | |  |
| aCCI |  | |  | 0.919 |  |  |  | 0.797 |
| ≤5 | 26 (0.00-54.44) | | |  |  | 77 (28.94-125.06) | |  |
| >5 | 31 (0.00-69.41) | | |  |  | 48 (8.39-87.61) | |  |
| Comorbidity |  | |  | 0.473 |  |  |  | 0.206 |
| No | 32 (0.00-103.34) | | |  |  | 87 (59.18-114.82) | |  |
| Yes | 23 (6.08-39.92) | | |  |  | 45 (28.80-61.20) | |  |
| Tumor location |  | |  | 0.143 |  |  | | 0.122 |
| Left lung | 70 (0.00-180.51) | | |  |  | 70 (7.28-132.72) | |  |
| Right lung | 31 (17.51-44.50) | | |  |  | 50 (14.39-85.61) | |  |
| T-stage |  | |  | 0.379 |  |  |  | 0.212 |
| T1 | 13 (7.95-18.05) | | |  |  | 48 (1.34-94.66) | |  |
| T2 | 32 (0.00-81.37) | | |  |  | 77 (25.89-128.11) | |  |
| T3 | 31 (0.00-66.15) | | |  |  | 46 (0.00-102.74) | |  |
| N-stage |  | |  | 0.159 |  |  |  | 0.156 |
| N0 | 31 (19.91-42.09) | | |  |  | 50 (21.80-78.21) | |  |
| N1 | 135 (-) | | |  |  | 135 (-) | |  |
| TNM-stage |  | |  | 0.555 |  |  |  | 0.746 |
| I | 16 (9.15-22.85) | | |  |  | 48 (0.16-95.84) | |  |
| IIA | 50 (0.00-115.08) | | |  |  | 50 (0.00-115.08) | |  |
| IIB | 31(0.00-101.56) | | |  |  | 69 (3.43-134.57) | |  |
| SIRI |  | |  | 0.088 |  |  |  | 0.207 |
| ≤0.79 | 11 (8.43-13.57) | | |  |  | 37 (16.49-57.51) | |  |
| >0.79 | 32 (0.00-92.61) | | |  |  | 70 (30.09-109.91) | |  |
| NLR |  | |  | 0.115 |  |  |  | 0.021 |
| ≤2.63 | 68 (0.00-155.18) | | |  |  | 101 (44.52-157.48) | |  |
| >2.63 | 26 (11.09-40.91) | | |  |  | 37 (19.97-54.03) | |  |
| PLR |  | |  | 0.018 |  |  |  | 0.006 |
| ≤150.36 | 68 (10.88-125.12) | | |  |  | 101 (45.42-156.58) | |  |
| >150.36 | 16 (3.27-28.73) | | |  |  | 31 (16.82-45.18) | |  |
| PNI |  | |  | 0.304 |  |  |  | 0.104 |
| ≤41.1 | 23 (0.00-99.23) | | |  |  | 37 (0.00-99.37) | |  |
| >41.1 | 31 (14.85-47.15) | | |  |  | 50 (11.23-88.77) | |  |
| CAR |  | |  | 0.071 |  |  |  | 0.026 |
| ≤0.91 | 32 (0.00-80.74) | | |  |  | 70 (32.53-107.47) | |  |
| >0.91 | 10 (0.00-25.10) | | |  |  | 12 (0.00-44.41) | |  |
| LCR |  | |  | 0.337 |  |  |  | 0.222 |
| ≤0.03 | 23 (0.00-56.36) | | |  |  | 37 (0.00-101.16) | |  |
| >0.03 | 32 (0.00-67.46) | | |  |  | 70 (38.08-101.92) | |  |
| RBC (10^12/L) |  | |  | 0.156 |  |  |  | 0.316 |
| ≤4.09 | 68 (0.10-135.91) | | |  |  | 70 (27.93-112.07) | |  |
| >4.09 | 16 (7.80-24.20) | | |  |  | 48 (27.19-68.81) | |  |
| Hb (g/L) |  | |  | 0.116 |  |  |  | 0.137 |
| ≤130 | 32 (0.00-85.58) | | |  |  | 70 (19.50-120.50) | |  |
| >130 | 18 (0.48-35.53) | | |  |  | 36 (28.05-43.95) | |  |
| Time from diagnosis to SBRT (days) |  | | | 0.421 |  |  | | 0.174 |
| ≤232 | 31 (19.00-43.00) | | |  |  | 70 (36.96-103.04) | |  |
| >232 | 32 (0.00-85.68) | | |  |  | 32 (0.00-85.68) | |  |
| Size (cm) |  | |  | 0.275 |  |  |  | 0.197 |
| ≤6.4 | 25 (11.19-38.81) | | |  |  | 37 (18.23-55.77) | |  |
| >6.4 | 70 (13.77-126.23) | | |  |  | 101 (56.49-145.51) | |  |
| PTV (cm^3^) |  | |  | 0.153 |  |  |  | 0.032 |
| ≤57.9 | 68 (0.00-145.88) | | |  |  | 87 (58.71-115.29) | |  |
| >57.9 | 25 (11.43-38.57) | | |  |  | 37 (20.80-53.20) | |  |
| BED (Gy) |  | | | 0.951 |  |  | | 0.934 |
| ≤100 | 26 (0.00-72.41) | | |  |  | 69 (37.03-100.93) | |  |
| >100 | 31 (14.28-47.72) | | |  |  | 48 (0.00-106.15) | |  |
| Systemic therapy |  | |  | 0.951 |  |  |  | 0.732 |
| Yes | 10.0 (0.00-39.33) | | |  |  | 45.0 (0.00-107.34) | |  |
| No | 31.0 (20.46-41.54) | | |  |  | 50.0 (22.64-77.36) | |  |
| PFS: progression-free survival; OS: overall survival; BMI: body mass index; aCCI: Age-adjusted Charlson Comorbidity Index; SIRI: systemic inflammation response index; NLR: neutrophil to lymphocyte ratio; PLR: platelet to lymphocyte ratio; PNI: prognostic nutritional index; CAR: C-reactive protein to albumin ratio; LCR: lymphocyte to C-reactive protein ratio; RBC: red blood cell count; Hb: hemoglobin; PTV: planning tumor volume; BED: biological effective dose | | | | | | | | |
