# Supplementary material for: Efficacy and safety of stereotactic radiotherapy on elderly patients with stage I-II central non-small cell lung cancer
Source: Front Oncol. 2024 May 13;14:1235630. doi: 10.3389/fonc.2024.1235630 (PMC11128597; doi:10.3389/fonc.2024.1235630)
Supplement: Supplementary file 3 [file Table_3.docx]

| Supplementary table 3 Univariate analysis of factors affecting cancer-specific death. | | | | | | | | |  |
| --- | --- | --- | --- | --- | --- | --- | --- | --- | --- |
| Variables |  | | Cancer-specific mortality | | | | | |  |
|  | 5-year mortality (95%CI） | | | |  | SHR (95%CI) | | P value | |
| Age (years)  ≤80  >80 |  | | |  |  |  |  | 0.620 | |
|  | 38.04 (21.84-54.11) | | | |  | Ref | |  | |
|  | 62.50 (17.35-88.15) | | | |  | 1.29 (0.48-3.43) | |  | |
| Gender | |  | |  |  |  |  | 0.610 | |
| Female | 50.00 (2.26-88.10) | | | |  | Ref | |  | |
| Male | 41.88 (25.93-57.06) | | | |  | 0.62 (0.10-3.81) | |  | |
| BMI (kg/m^2^) | |  | |  |  |  |  | 0.000 | |
| ≤22.77 | 23.11 (9.15-40.77) | | | |  | Ref | |  | |
| >22.77 | 77.31 (41.80-92.69) | | | |  | 3.96 (1.81-8.68) | |  | |
| Smoking index |  | | |  |  |  |  | 0.220 | |
| ≤1100 | 39.42 (22.62-55.82) | | | |  | Ref | |  | |
| >1100 | 55.56 (17.08-82.24) | | | |  | 1.65 (0.74-3.65) | |  | |
| Performance status |  | | |  |  |  |  | 0.170 | |
| 0/1 | 36.42 (20.21-52.84) | | | |  | Ref | |  | |
| 2 | 67.50 (23.24-89.96) | | | |  | 1.86 (0.77-4.48) | |  | |
| aCCI |  | | |  |  |  |  | 0.180 | |
| ≤5 | 33.03 (12.77-55.08) | | | |  | Ref | |  | |
| >5 | 50.00 (28.41-68.26) | | | |  | 1.75 (0.77-3.97) | |  | |
| Comorbidity |  | | |  |  |  |  | 0.019 | |
| No | 19.79 (4.47-42.99) | | | |  | Ref | |  | |
| Yes | 56.49 (35.03-73.27) | | | |  | 2.75 (1.18-6.42) | |  | |
| Location |  | | |  |  |  | | 0.810 | |
| Left lung | 43.75 (18.86-66.38) | | | |  | Ref | |  | |
| Right lung | 43.05 (23.08-61.61) | | | |  | 1.11 (0.49-2.53) | |  | |
| T-stage |  | | |  |  |  |  |  | |
| T1 | 48.61 (6.23-82.91) | | | |  | Ref | |  | |
| T2 | 41.99 (21.82-60.98) | | | |  | 1.04 (0.30-3.66) | | 0.950 | |
| T3 | 43.06 (13.95-69.73) | | | |  | 1.54 (0.40-5.84) | | 0.530 | |
| N-stage |  | | |  |  |  |  | 0.490 | |
| N0 | 43.82 (27.66-58.89) | | | |  | Ref | |  | |
| N1 | 33.33 (0.14-83.15) | | | |  | 0.43 (0.04-7.71) | |  | |
| TNM-stage |  | | |  |  |  |  |  | |
| I | 40.53 (17.28-62.84) | | | |  | Ref | |  | |
| IIA | 50.00 (16.25-76.77) | | | |  | 0.95 (0.37-2.48) | | 0.920 | |
| IIB | 40.83 (15.67-64.86) | | | |  | 1.13 (0.47-2.71) | | 0.780 | |
| SIRI |  | | |  |  |  |  | 0.530 | |
| ≤0.79 | 85.71 (0.00-99.96) | | | |  | Ref | |  | |
| >0.79 | 39.30 (23.23-55.02) | | | |  | 0.72 (0.26-1.99) | |  | |
| NLR |  | | |  |  |  |  | 0.340 | |
| ≤2.63 | 37.61 (14.51-60.93) | | | |  | Ref | |  | |
| >2.63 | 46.57 (26.23-64.64) | | | |  | 1.45 (0.68-3.13) | |  | |
| PLR |  | | |  |  |  |  | 0.089 | |
| ≤150.36 | 34.74 (16.07-54.26) | | | |  | Ref | |  | |
| >150.36 | 53.53 (27.85-73.68) | | | |  | 1.94 (0.90-4.17) | |  | |
| PNI |  | | |  |  |  |  | 0.005 | |
| ≤41.1 | 50.00 (12.53-79.35) | | | |  | Ref | |  | |
| >41.1 | 41.57 (24.55-57.78) | | | |  | 0.38 (0.19-0.74) | |  | |
| CAR |  | | |  |  |  |  | 0.002 | |
| ≤0.91 | 39.19 (23.09-54.96) | | | |  | Ref | |  | |
| >0.91 | 66.67 (12.22-92.48) | | | |  | 3.65 (1.61-8.24) | |  | |
| LCR |  | | |  |  |  |  | 0.066 | |
| ≤0.03 | 57.14 (13.08-85.73) | | | |  | Ref | |  | |
| >0.03 | 40.34 (23.80-56.33) | | | |  | 0.42 (0.17-1.06) | |  | |
| RBC (10^12/L) |  | | |  |  |  |  | 0.380 | |
| ≤4.09 | 30.42 (11.88-51.43) | | | |  | Ref | |  | |
| >4.09 | 53.73 (30.55-72.22) | | | |  | 1.40 (0.65-3.01) | |  | |
| Hb (g/L) |  | | |  |  |  |  | 0.400 | |
| ≤130 | 37.19 (20.02-54.43) | | | |  | Ref | |  | |
| >130 | 57.14 (23.57-80.52) | | | |  | 1.44 (0.62-3.32) | |  | |
| Time from diagnosis to SBRT (days) |  | | |  |  |  | | 0.820 | |
| ≤232 | 43.50 (26.94-58.96) | | | |  | Ref | |  | |
| >232 | 40.00 (3.06-78.61) | | | |  | 1.18 (0.28-4.94) | |  | |
| Size (cm) |  | | |  |  |  |  | 0.410 | |
| ≤6.4 | 48.04 (29.36-64.50) | | | |  | Ref | |  | |
| >6.4 | 29.29 (6.04-58.44) | | | |  | 0.71 (0.31-1.61) | |  | |
| PTV (cm^3^) |  | | |  |  |  |  | 0.070 | |
| ≤57.9 | 19.35 (4.37-42.23) | | | |  | Ref | |  | |
| >57.9 | 56.48 (34.96-73.31) | | | |  | 2.09 (0.94-4.65) | |  | |
| BED (Gy) |  | | |  |  |  |  | 0.240 | |
| ≤100 | 48.33 (23.90-69.11) | | | |  | Ref | |  | |
| >100 | 38.68 (19.04-58.04) | | | |  | 0.64 (0.30-1.35) | |  | |
| ODR: ; BMI: body mass index; aCCI: Age-adjusted Charlson Comorbidity Index; SIRI: systemic inflammation response index; NLR: neutrophil to lymphocyte ratio; PLR: platelet to lymphocyte ratio; PNI: prognostic nutritional index; CRP/Alb: C-reactive protein to albumin ratio; LCR: lymphocyte to C-reactive protein ratio; RBC: red blood cell count; Hb: hemoglobin; PTV: planning tumor volume; BED: biological effective dose | | | | | | | | | |
