# Supplementary figures and images for: Efficacy and safety of stereotactic radiotherapy on elderly patients with stage I-II central non-small cell lung cancer
Source: Front Oncol. 2024 May 13;14:1235630. doi: 10.3389/fonc.2024.1235630 (PMC11128597; doi:10.3389/fonc.2024.1235630)

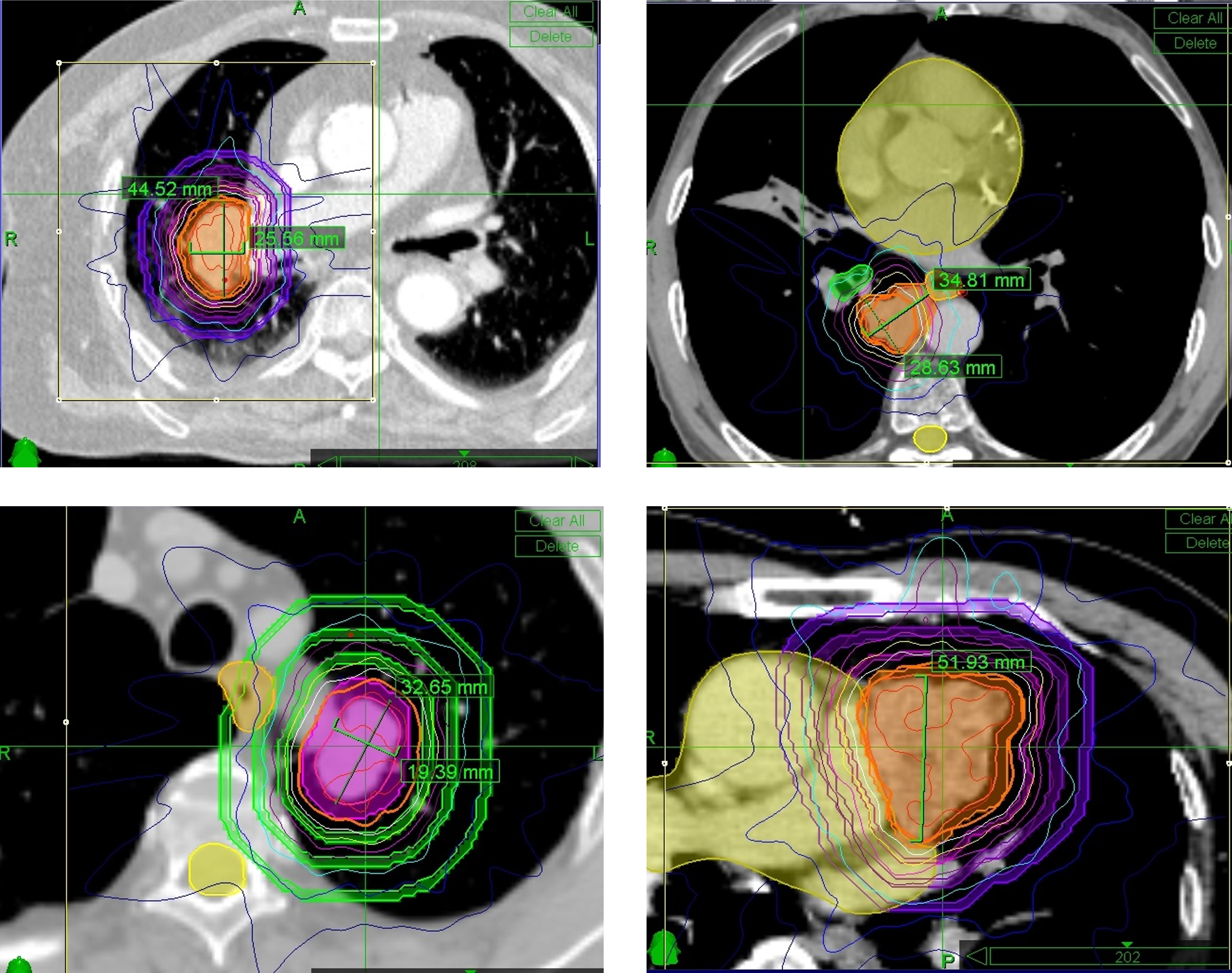

Supplement: Supplementary Figure 1 — CT images of several typical central lung cancer patients. [file Image_1.jpeg]
